# Effectiveness of instrumented gait analysis in interdisciplinary interventions on parents’ perception of family-centered service and on gross motor function in children with cerebral palsy: a randomized controlled trial

**DOI:** 10.1186/s12887-020-02315-2

**Published:** 2020-09-01

**Authors:** Christina Esmann Fonvig, Helle Mätzke Rasmussen, Søren Overgaard, Anders Holsgaard-Larsen

**Affiliations:** 1 The Orthopaedic Research Unit, Department of Orthopaedic Surgery and Traumatology, Odense University Hospital, Odense, Denmark; 2 Department of Clinical Research, University of Southern Denmark, Odense, Denmark

**Keywords:** Cerebral palsy, Family-centered service, Instrumented gait analysis, GMFM-66

## Abstract

**Background:**

Children with cerebral palsy often exhibit an altered gait pattern; however, it is uncertain whether the use of an instrumented gait analysis in interdisciplinary interventions affects the perceived experience of family-centered service (FCS) and/or gross motor function. The aim of this study is **t**o investigate whether individually tailored interdisciplinary interventions, based on an instrumented gait analysis report, has a superior effectiveness on perceived FCS and gross motor function in children with cerebral palsy, compared to ‘care as usual’ without the use of instrumented gait analysis. Furthermore, to investigate potential associations between perceived FCS and gross motor function improvement with the goal of improving future therapy on gross motor function.

**Method:**

This is a sequel analysis on tertiary outcome measures from a prospective, single blind, randomized, parallel group study including two groups of 30 children aged 5–8 years with spastic cerebral palsy at Gross Motor Function Classification System levels I-II (*n* = 60). The intervention group underwent a three-dimensional gait analysis, from which a clinical report was written with recommendations on interdisciplinary interventions, such as physical therapy, orthopedic surgery, orthotics or spasticity management.

To assess effectiveness on perceived FCS and gross motor function, at baseline, 26 weeks and 52 weeks, the five domains in the Measure of Processes of Care (MPOC-20) (*Enabling and partnership*, *Providing general information*, *Providing specific information about the child*, *Respectful and supportive service,* and *Coordinated and comprehensive care*) and the Gross Motor Function Measurement (GMFM-66) were used as outcome measures.

**Results:**

No significant differences in between-group change scores in any of the five MPOC-20 domains were observed (*p* = 0.40–0.97). In favor of the intervention group a significantly higher between-group change score in GMFM-66 (mean difference: 3.05 [95%CI: 1.12–4.98], *p* = 0.003) after 52 weeks was observed.

**Conclusion:**

The addition of an instrumented gait analysis report to ‘care as usual’ did not improve the parents’ perceptions of FCS in treatment of children with cerebral palsy. However, superior improvement in the GMFM-66 was observed in the intervention group, suggesting meaningful gross motor function improvement.

**Trial registration:**

Clinical Trials, NCT02160457. Registered June 10th 2014.

## Background

Cerebral palsy (CP) is an umbrella term for a range of conditions caused by a non-progressive injury in the developing fetal or infant brain, resulting in permanent disorders of the development of movement and posture, causing activity limitations [1]. With a prevalence of 2–3 per 1000 live births, CP is the most common disabling impairment amongst children [2, 3].

Although 50–80% of children with CP are capable of walking independently, gait is usually affected as a result of musculoskeletal deformity, abnormal muscle tone, inadequate balance and impaired motor control [4]. As a result, the following impairments may be observed: drop foot during swing phase, plantarflexion of foot in stance, leg length discrepancy or restricted joint movement due to muscle contracture [5].

To monitor and improve the quality of healthcare for children with CP, standardized clinical examinations are offered throughout childhood as a means of early detection of complications [6]. The hospital pediatric departments offer interdisciplinary consultations, where children with CP, their families and the local healthcare team, consisting of professionals from the municipal and regional healthcare systems, meet and agree on future surveillance, coordinate common goals and plan interdisciplinary interventions for the child [6]. However, the current standard care does not include systematic evaluation of the child’s gait-related impairment.

Family-centered service (FCS) is considered best practice in providing healthcare services to children and their families, and is associated with improved parent satisfaction within healthcare services, strengthening of family empowerment, and improved development and functioning of the child [7, 8]. One possible way to support an FCS approach in children with CP may be to provide the family and the healthcare personnel with a detailed description of the child’s gait-related impairments along with specific recommendations regarding tailored interventions, thus warranting the involvement of the family in the planning of their child’s treatment.

However, in the current standard care for children with CP, which does not include systematic evaluation of the child’s gait-related impairments, it is uncertain what the family’s overall perception of FCS is and to which degree interventions are agreed upon in collaboration with the child’s parents at interdisciplinary consultations.

As a means of clarifying the intervention needs of a child with CP, an evaluation of gait-related impairments by an instrumented gait analysis (IGA) can be used to provide objective and valid measures of gait. This analysis identifies features affecting gross motor function and underlying neuro-musculoskeletal impairments [4].

Studies show that surgeons follow the IGA recommendations 42–97% of the time [9–11] and in cases where surgery had already been decided, the addition of an IGA has been shown to change 40–89% of surgical treatment plans [9, 12–14]. Thus, IGA is considered a valuable tool in clinical decision making. However, while it has not been previously investigated, it is also reasonable to expect that the addition of an IGA report could support an FCS approach, as the IGA report supplies the families with detailed information on their child’s gait as well as recommendations (in layman’s terms) for interventions. Thus, this report could allow them to participate in the planning and tailoring of specific interdisciplinary interventions in conjunction with physiotherapists, orthopedic surgeons, neuropediatricians and other healthcare professionals.

Gross motor function can be used as a proxy measure for overall improvement during intervention [15]. A potential association between improvement in parents’ perceptions of the FCS they and their children received during the study period and the child’s gross motor improvement could indicate that gross motor function is related to the parents’ experiences of, and involvement in, the processes of care. Thus, an association could serve as an indicator for healthcare personnel allowing them to detect families who may benefit from enhanced FCS.

This study aimed to test the hypothesis that the use of an IGA report in the decision making of interdisciplinary interventions for children with CP, has a superior effectiveness on the parents’ perceived experience of FSC and on the child’s gross motor function compared to ‘care as usual’, without the use of an IGA report.

Furthermore, to test if potential associations between perceived experience of FCS and gross motor function exists with the perspective of detecting components of the FCS experience worthwhile targeting to potentially improve gross motor function.

## Methods

This study is a sequel analysis (on tertiary outcome measures) of a randomized controlled trial based on a dataset powered for a different question [16, 17]. The study is approved by the Committee for Medical Research Ethics in the Region of Southern Denmark (S-20120162) and the Danish Data Protection Agency (2008-58-0035) and is compliant with the Declaration of Helsinki. Tertiary outcome measures for the present analysis were a priori defined at ClinicalTrials.gov (NCT02160457) [17]. Data collection was performed August 2014 to July 2017. Reporting followed the CONSORT statement for pragmatic trials [18].

One hundred and sixty children with CP from the Regions of Southern Denmark and North Denmark were screened for eligibility and invited to participate, of whom 60 were randomized to either the experimental or the control group after baseline assessment. Randomization and allocation is described in detail elsewhere [16]. In brief, the allocation sequence was computer generated by a researcher with no other involvement in the study. The allocation sequence was concealed in sequentially numbered, opaque, sealed envelopes. After completion of baseline assessment, the project manager of the trial (HMR) opened the envelope and informed the parents and the local team about the allocation [16, 17].

Inclusion criteria were children aged five to 8 years with a diagnosis of spastic CP at Gross Motor Function Classification System (GMFCS) level I-II [19]. Exclusion criteria were 1) orthopedic surgery within 52-weeks prior to baseline assessment, 2) injection with botulinum toxin type A within 12-weeks prior to baseline assessment, 3) inability to participate in examination, or 4) lack of ability to speak and understand Danish. Parents signed informed consent following the receipt of verbal and written information at the Motion Analysis Laboratory at Odense University Hospital [16].

Administration of data collection was performed blinded. The participants (children with CP and their parents) and the local health teams were unblinded to the intervention.

### Intervention

The interventions under investigation were: 1) individually tailored interdisciplinary intervention based on recommendations from measures performed as part of standard care as well as from an IGA report and 2) individually tailored interdisciplinary intervention based on clinical examinations WITHOUT an IGA report (‘care as usual’).

The IGA was performed at baseline, and the report was subsequently sent to the families as well as the treating healthcare personnel so that the information was available at the following interdisciplinary consultations held at the pediatric departments.

The interdisciplinary interventions recommended in the IGA report have been described in detail elsewhere [17]; but in brief they were divided into the following four categories: (1) physical therapy, (2) orthotics, (3) spasticity management, and (4) orthopedic surgery. The recommended interventions were provided by the child’s local healthcare team regardless of allocation group. Due to the study’s pragmatic approach, interventions made at multiple departments and/or clinics were not standardized, nor was adherence to the recommended interventions a requirement. For an anonymized example of an instrumented gait analysis report, including interdisciplinary interventions, see Additional file 1: IGA report.

### Outcome measures

Height and weight, used for the calculation of Body Mass Index Standard Deviation Score (BMI SDS), were measured at baseline. CP subtype was collected from the local health teams.

To assess the degree of perceived FCS, responses to the Danish version of the Measure of Processes of Care (MPOC-20) questionnaire [20], were obtained at baseline, 26 weeks and 52 weeks post start of intervention. The primary endpoint of the present sequel analysis was the change-score in the perception of FSC from baseline to 52 weeks.

The MPOC-20 is a self-report measure of the parents’ perceptions of the extent to which the health services their child receive are family-centered [21]. The 20 items cover issues of five selected domains; *Enabling and partnership*, *Providing general information*, *Providing specific information about the child*, *Respectful and supportive service* and *Coordinated and comprehensive care.* This tool is widely used in pediatric rehabilitation to evaluate FCS [7]. The clinometric properties of the MPOC-20 have shown solid internal consistency, test-retest reliability and good evidence of construct validity [21].

To assess gross motor function, the Gross Motor Function Measure (GMFM-66), a gold standard measure of functional ability in the area of CP [22], was performed at baseline and at 52 weeks post start of intervention. A minimum requirement of 13 relevant items of the GMFM-66 were used for a valid calculation of the GMFM-66 using the Gross Motor Ability Estimator Scoring Software [23]. The GMFM-66 is valid and reliable (ICC = 0.99) [23] and is sensitive enough to document clinically meaningful improvement, including responsiveness [24].

### Statistical analysis

Study power considerations for this tertiary analysis were evaluated a posteriori and showed that if at least 56 participants completed the trial, it would yield a power between 65 and 92% to detect a between-group difference (*p* < 0.05) of 1 MPOC point with a SD of 1.19–1.82 on the MPOC scale ranging from 0 to 7 (where higher scores indicate that the parents perceive that their needs are being better met). Since no anchor-based minimum clinically important difference (MCID) is available for the MPOC-20, the present study determined one MPOC point as being clinically relevant, as it would increase the scale score of the present sample to what has been seen in international references [7].

The statistical analysis was a priori defined at ClinicalTrials.gov (NCT02160457) [17] and was performed according to intention-to-treat and, due to low expectation of drop-out, with last-observation-carried-forward for missing observations. Descriptive statistics were summarized as appropriate. Evaluating superiority was estimated with a linear model, with between-group change differences as dependent variable and randomization group as independent variable, adjusted for relevant baseline score. Furthermore, categorical analyses investigating the risk difference (RD) between groups in scoring above an MCID for the MPOC-20 and GMFM-66 were used to calculate a possible Number Needed to Treat (NNT). Finally, associations between the MPOC-20 and GMFM-66 were evaluated using multiple linear regressions with A) the MPOC-20 domains at 52–week follow-up as the dependent variable, and the GMFM-66 change score at the 52–week follow-up as the independent variable, adjusted for baseline GMFM-66 score, sex, age, BMI SDS, CP spastic subtype, and GMFCS level, as well as with B) the GMFM-66 change score at the 52–week follow-up as the dependent variable and the MPOC-20 domains at the 52–week follow-up as the independent variable, adjusted for baseline MPOC-20 domain score, sex, age, BMI SDS, CP spastic subtype and GMFCS level. Effect sizes are reported as partial Eta2 for the independent variable.

Statistical analyses were performed using Stata/IC 14.2 or later for Windows (StataCorp LLC, College Station TX, USA). Results are presented with an alpha of 0.05 and a 95% confidence interval.

## Results

**Fig. 1:**
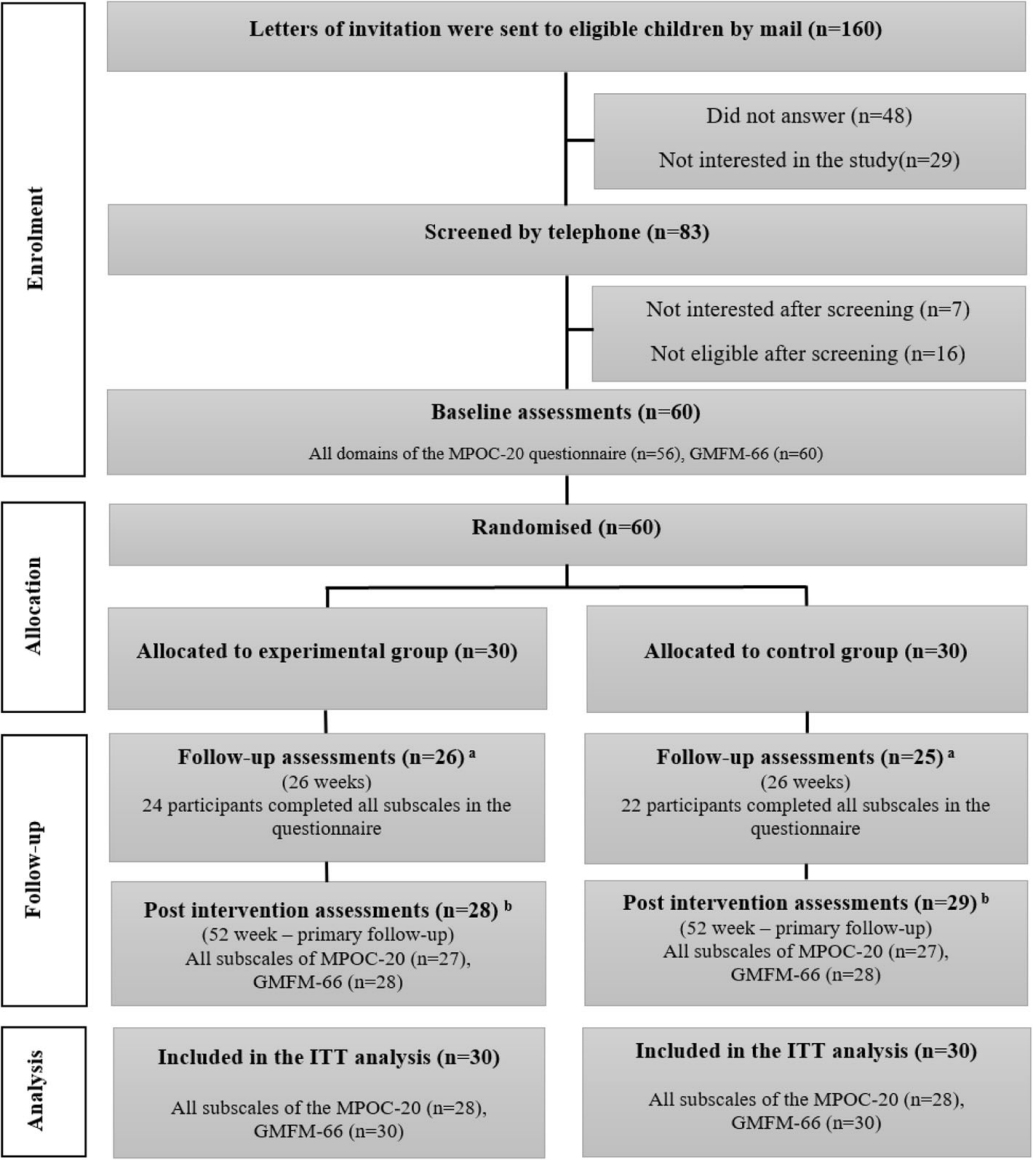
Flow diagram of participants in the study. Explanations: ^a^Three participants did not complete the MPOC-20 questionnaire. ^b^Six participants did not complete the MPOC-20 questionnaire. Four participants did not complete the GMFM-66 assessment

Of the 160 children invited to participate in this study, 83 were screened for eligibility, of which 60 were randomized to either the experimental group (*n* = 30) or the control group (*n* = 30). For the primary outcome, complete assessments were available from 56 participants at baseline, 46 participants at the 26-week follow-up, and 54 participants at the primary endpoint of 52 weeks (Fig. 1).

The 60 included children had a median age of 6 years and 10 months (Table 1). The CP subtypes were 43 children with unilateral spastic CP (experimental (*n* = 21) / control (*n* = 22)), and 17 children with bilateral spastic CP (experimental (*n* = 9) / control (*n* = 8)). GMFCS levels were 42 children at level I (experimental (*n* = 20) / control (*n* = 22)), and 18 children at level II (experimental (*n* = 10) / control (*n* = 8)) (Table 1).

**Table 1:** Demographics and baseline data of included children

	Experimental group *n* = 30	Control group *n* = 30
Sex *boys/girls*, n	21/9	18/12
CP spastic subtype *unilateral/bilateral*, n	21/9	22/8
GMFCS level *I/II*, n	20/10	22/8
Age *years, months*, median (IQR)	6y 6 m (2y 8 m)	6y 11 m (1y 10 m)
Height *m*	1.12 (0.08)	1.24 (0.11)
Weight *kg*, median (IQR)	22 (6)	21 (11)
BMI SDS	−0.32 (1.46)	−0.62 (1.42)
Measure of Processes of Care - 20 domains		
*enabling and partnership*	4.27 (1.80) ^a^	3.87 (1.82) ^a^
*providing general information*	3.40 (1.65) ^a^	2.95 (1.37) ^b^
*providing specific information about the child*	4.47 (1.78) ^a^	3.57 (1.66) ^b^
*respectful and supportive service*	4.64 (1.56) ^a^	4.45 (1.29) ^b^
*coordinated and comprehensive care*	5.06 (1.34) ^a^	4.63 (1.19) ^a^
Gross Motor Function Measure – 66	81.17 (8.19)	83.63 (8.51)

Values are reported as mean and standard deviation (SD) unless stated otherwise

Abbreviations: *CP* Cerebral Palsy, *GMFCS* Gross Motor Function Classification System, *BMI SDS* Body Mass Index Standard Deviation Score showing the BMI percentile and z-score [25], *n* number, *IQR* interquartile range, *m* meters, *kg* kilogram

Explanations: ^a^ *n* = 28, ^b^ *n* = 29

As previously stated [16], no serious adverse events were reported during the study period.

Opposed to the hypothesis, the present study found no superior effectiveness in FSC evaluated by MPOC-20 domains at 52-weeks follow-up (*Enabling and partnership*: 0.02 [95%CI: − 0.88 – 0.92], *p* = 0.97, *Providing general information*: -0.16 [95%CI: − 1.00 – 0.69], *p* = 0.71, *Providing specific information about the child*: -0.39 [95%CI: − 1.33 – 0.54], *p* = 0.40, *Respectful and supportive service*: 0.14 [95%CI: − 0.56 – 0.83], *p* = 0.70, *Coordinated and comprehensive care*: 0.04 [95%CI: − 0.62 – 0.69], *p =* 0.92) (Table 2). Furthermore, no between-group changes were observed at 26-weeks follow-up (*p*-values ranging from 0.26–0.97 for the five domains) (Table 2). Statistical analyses between weeks 26 and 52 were calculated post hoc and showed no significant difference when looking at within or between group changes (data not shown). The categorical analysis investigating the number of participants scoring above the a posteriori set MCID of 1 MPOC point at the 52-week follow-up, showed no significant differences between the two groups in any of the five domains (Table 3).

**Table 2:** Within- and between-group differences at the 26-week and 52-week follow-ups

	26-weeks follow-up (mean change from baseline)			*p-*value	Effect size (Eta^2^)	52-weeks follow-up (mean change from baseline)			*p-*value	Effect size (Eta^2^)
	EXPERIMENTAL (mean ± SE) (within-group)	CONTROL (mean ± SE) (within-group)	Between-group difference in change (mean [95% CI])			EXPERIMENTAL (mean ± SE) (within-group)	CONTROL (mean ± SE) (within-group)	Between-group difference in change (mean [95% CI])		
MPOC-20 domains†										
*enabling and partnership* ^*a*^	−0.03 ± 0.22	0.27 ± 0.30	− 0.17 [− 0.84–0.50]	0.43	0.004	− 0.14 ± 0.36	0.08 ± 0.39	0.02 [− 0.88–0.92]	0.97	0.000
*providing general information* ^*b*^	−0.06 ± 0.33	0.04 ± 0.33	0.09 [− 0.80–0.97]	0.83	0.001	−0.41 ± 0.33	0.03 ± 0.35	−0.16 [− 1.00–0.69]	0.71	0.002
*providing specific information about the child* ^*b*^	− 0.40 ± 0.28	0.06 ± 0.30	−0.10 [− 0.92–0.71]	0.26	0.001	−0.80 ± 0.43	0.18 ± 0.31	−0.39 [− 1.33–0.54]	0.40	0.014
*respectful and supportive service*^*b*^	0.23 ± 0.21	0.04 ± 0.24	0.03 [− 0.57–0.64]	0.97	0.000	−0.09 ± 0.27	−0.16 ± 0.26	0.14 [− 0.56–0.83]	0.70	0.003
*coordinated and comprehensive care* ^*a*^	0.16 ± 0.20	0.09 ± 0.19	0.13 [− 0.42–0.69]	0.79	0.004	−0.11 ± 0.21	−0.05 ± 0.29	0.04 [− 0.62–0.69)]	0.92	0.000
GMFM-66 ^c^	–	–	–	–		3.32 ± 0.84*	0.16 ± 0.48	3.05 [1.12–4.98]	0.003	0.145

Between-group differences are adjusted for relevant baseline score

Abbreviations: *MPOC-20* Measure of Processes of Care, *GMFM-66* Gross Motor Function Measure

Explanations: †Primary outcome measure, *Within-group difference in change score *p* < 0.001, ^a^ Experimental group, *n* = 28 and control group, *n* = 28, ^b^ Experimental group, *n* = 28 and control group, *n* = 29, ^c^ Experimental group, *n* = 30 and control group, *n* = 30

**Table 3:** Categorical analysis of participants scoring above an MCID of 1 MPOC-20 point

	EXPERIMENTAL	CONTROL	RD	[95%CI]	*p*-value
MPOC-20 domains					
*enabling and partnership*	6/28	7/28	−0.04	[− 0.26–0.19]	0.75
*providing general information*	7/28	4/29	0.11	[−0.09–0.32]	0.28
*providing specific information about the child*	4/28	8/29	−0.13	[−0.34–0.07]	0.21
*respectful and supportive service*	6/28	2/29	0.15	[−0.03–0.32]	0.11
*coordinated and comprehensive care*	3/28	5/28	−0.07	[−0.07–0.11]	0.44

Values reported as amount in numbers, risk difference (RD), and 95% confidence interval (CI)

Abbreviations: *MCID* Minimal Clinically Important Difference, *MPOC-20* Measure of Processes of Care

A statistically significant between-group change score in the GMFM-66 in favor of the experimental group (3.05 [95%CI: 1.12–4.98], *p* = 0.003) was observed at the 52-week follow-up (Table 2). On the individual level, the number of children that had a gross motor function improvement above the defined MCID of 1.7 [24] was 16/30 (53%) in the experimental group and 6/30 (20%) in the control group; this difference was in favor of the experimental group (RD = 0.33 [95%CI: 0.1–0.56], *p* = 0.004) with an NNT of 3.

The association analyses showed no indication of either a uni- or bidirectional relationship between the parents’ general perceived experiences of FCS over the past year and the child’s gross motor function improvement (Tables 4 and 5).

**Table 4:** Associations between MPOC-20 and GMFM-66

Dependent variable: MPOC-20 52-weeks follow-up domain scores	Independent variable: ΔGMFM_**0–52**_ score	(mean ± SE)	[95%CI]	*p-*value	Effect size (Eta^2^)
*enabling and partnership*		0.10 ± 0.05	[− 0.01–0.20]	0.066	0.044
*providing general information*		0.01 ± 0.06	[− 0.10–0.12]	0.816	0.001
*providing specific information about the child*		0.002 ± 0.06	[− 0.12–0.12]	0.975	0.000
*respectful and supportive service*		0.07 ± 0.04	[−0.02–0.15]	0.106	0.032
*coordinated and comprehensive care*		0.05 ± 0.04	[−0.03–0.13]	0.214	0.016

Dependent variable: MPOC-20 domains at the 52-week follow-up

Independent variable: GMFM-66 change score at the 52-week follow-up

Adjusted for baseline GMFM-66 score, sex, age, BMI SDS, CP spastic subtype, and GMFCS level

Abbreviations: *GMFM-66* Gross Motor Function Measure, *GMFCS* Gross Motor Function Classification System, *MPOC-20* Measure of Processes of Care, *CI* confidence intervals, *SE* standard error

**Table 5:** Associations between MPOC-20 and GMFM-66

Dependent variable: ΔGMFM_**0–52**_ score	Independent variable: MPOC-20 52-weeks follow-up domain scores	(mean ± SE)	[95%CI]	*p-*value	Effect size (Eta^2^)
	***enabling and partnership***	0.66 ± 0.39	[−0.14–1.45]	0.103	0.070
	***providing general information***	0.27 ± 0.41	[−0.56–1.10]	0.511	0.003
	***providing specific information about the child***	−0.27 ± 0.41	[−1.10–0.55]	0.510	0.002
	***respectful and supportive service***	0.61 ± 0.44	[− 0.28–1.50]	0.175	0.055
	***coordinated and comprehensive care***	0.23 ± 0.34	[− 0.46–0.91]	0.512	0.034

Dependent variable: GMFM-66 change score at the 52-week follow-up

Independent variable: MPOC-20 domains at the 52-week follow-up

Adjusted for relevant MPOC-20 domain score, sex, age, BMI SDS, CP spastic subtype, and GMFCS level

Abbreviations: *GMFM-66* Gross Motor Function Measure, *GMFCS* Gross Motor Function Classification System, *MPOC-20* Measure of Processes of Care, *CI* confidence intervals, *SE* standard error

## Discussion

In this secondary analysis on tertiary data from a randomized controlled trial, we observed no superior effectiveness in parents’ perceived experience of FSC at the 52-week follow-up (primary endpoint) or the 26-week follow-up, when providing an IGA report as part of the interdisciplinary interventions for children with CP. Hence, the results cannot support an implementation of IGA as part of the standard care for children with CP at GMFCS level I–II solely with the intention of improving perceptions of FCS. However, a superior improvement in gross motor function was observed in the experimental group, suggesting that the use of an IGA in addition to ‘care as usual’, may benefit gross motor function. However, these findings need to be confirmed in a priori designed studies with the specific purpose of detecting changes in gross motor function.

Although studies have shown, that the use of IGA can alter the clinical decisions about planned surgical interventions [9, 26], the present study, on a sample of children with CP showed that it did not alter the parents’ perceived experiences of FCS.

The idea of adding an IGA report with specific recommendations for interventions for the child was to supply the parents, as well as the healthcare personnel, with a detailed, objective explanation of the impairments present in order to improve the observed gait difficulties. However, to comply with current clinical practice, the present pragmatic study design did not include a thorough oral explanation of the IGA report itself; thus, we do not know whether the parents and/or healthcare personnel understood or even read the report.

Generally, all MPOC-20 domains observed in the present study were evaluated one point below international reference scores [7], indicating generally low perceptions of FCS in the treatment of children with CP in Denmark. Therefore, there is room for improvement for the present healthcare personnel using an FCS approach, and the lack of effectiveness observed in the present study cannot be attributed to a ceiling effect. A previous study investigating parents’ perceived experiences of FCS in three municipalities in Denmark [27] reported similar low scores in three of the five domains. This emphasizes the necessity to involve the families in the decision-making process for their child’s care and also highlights the parents’ strong desire for both specialized knowledge about children with CP in general and specific knowledge concerning their own child.

The present lack of change in the perception of FCS could be because gait function was predefined without the involvement of the families. The parents may have preferred to focus on other areas, e.g., general health, self-care, communication, or caregiver issues [15]. Thus, the inclusion of an IGA report with the intention of improving gait may not necessarily have been a prioritized need of the families.

A clinically relevant between-group change score in the GMFM-66 was detected in favor of the experimental group (3.05 [95%CI: 1.08–5.02] *p* = 0.003) at the 52-week follow-up, resulting in an NNT of 3. However, this is in divergence to the main study of the present trial, which found no improvement in overall gait function [16]. The calculation of the GMFM-66 in the present study was based upon a minimum of items; consequently, the results should be interpreted with caution, taking into consideration the risk of a Type I error. Furthermore, as with most clinical trials, a potential selection bias could have influenced the results.

The present study found no association between the measured change score in the GMFM-66 and the follow-up score in the MPOC-20. Hence, the hypothesis that an association between these items could be an indicator of families that would benefit from enhanced FCS as a means of improving the child’s gross motor function could not be confirmed. However, the present results from this singular, tertiary trial on the potential improvement in perceived FCS by adding IGA to the decision making should not be considered an exhaustive conclusion on the topic.

### Strengths and limitations

The external validity of the study is strong due to the pragmatic study design with generalizability to the Danish population of 5–8-year-old children with CP at GMFCS level I–II. However, the results may not be generalizable to older children with CP or to children with more profound disabilities. Furthermore, the risk of selection bias is unknown, as data on non-participants (*n* = 84) is unavailable. This should be taken into account.

The present study design did not allow the parents to attend a consultation where they were individually informed about the content of the IGA report by the researchers who evaluated and recommended the interventions. Doing so could potentially have helped the parents understand the contents of the report, thereby providing them with specific information about their child and increasing their ability to be critically involved in the planning of their child’s treatment. No data of the parents’ psychological health was collected, and thus it could not be evaluated.

Another limitation is that an anchor-based MCID for the MPOC-20 is not available, and, consequently, the cut-off point of one MPOC-20 point may be considered arbitrary despite being based upon reference values in the literature [7]. Furthermore, three out of five post hoc power analyses for MPOC-20 domains were underpowered for effects in between-group FCS change. Additionally, statistical results were not corrected for multiple testing, as the study is based on tertiary outcomes [28]. However, no statistical differences in between-group change scores were observed, and the observed wide 95% confidence intervals demonstrated with a clear statistical certainty that the intervention does not provide superior effectiveness on parents’ perception of FCS. A primary analysis on an anchor-based MCID for the MPOC-20 is needed to evaluate clinically relevant changes in perception of FCS over time.

Finally, as the present study is not powered to detect a between-group difference in GMFM-66, it is necessary to confirm the above-mentioned results in an a priori designed study with the primary purpose to investigate the use of an IGA report in interdisciplinary intervention on gross motor function improvement.

## Conclusions

In this secondary analysis on tertiary data from a randomized controlled trial, the addition of an IGA in an individually tailored interdisciplinary intervention showed no superior effectiveness in improving the parents’ perceived experiences of FCS in a relatively young group of children with CP at GMFCS level I-II. The results can therefore not support an implementation of IGA as part of standard care with the sole intention of improving the parents’ perception of FCS. However, the addition of IGA to ‘care as usual’ may benefit the child’s gross motor function. These findings need to be confirmed in a priori designed studies with the specific purpose of detecting change in gross motor function. The study could not detect an association between perception of FCS and gross motor function.

In perspective, the results from the present study warrant further investigation into the reasons behind the relative parental dissatisfaction with FCS, and into which steps can be addressed in the clinical decision making process to improve parents’ perception of FCS when planning interdisciplinary approaches for improved gait function. This would provide clinical knowledge about how to ensure that the parents’ needs for specialized information and involvement are more appropriately met in the future.

## Supplementary information

**Additional file 1.**

## Data Availability

The datasets used and/or analyzed during the current study are available from the corresponding author on reasonable request.

## Abbreviations

BMI SDS: Body Mass Index Standard Deviation Score
CP: Cerebral palsy
FCS: Family-centered service
GDI: Gait Deviation Index
GMAE-2: Gross Motor Ability Estimator Scoring Software
GMFCS: Gross Motor Function Classification System
GMFM-66: Gross Motor Function Measure
MCID: Minimum clinically important difference
MPOC-20: Measure of Processes of Care
IGA: Three-dimensional instrumented gait analysis
