# Supplementary material for: Effectiveness of instrumented gait analysis in interdisciplinary interventions on parents’ perception of family-centered service and on gross motor function in children with cerebral palsy: a randomized controlled trial
Source: BMC Pediatr. 2020 Sep 1;20:411. doi: 10.1186/s12887-020-02315-2 (PMC7461340; doi:10.1186/s12887-020-02315-2)
Supplement: Supplementary file 1 — Additional file 1. [file 12887_2020_2315_MOESM1_ESM.pdf]

Gait analysis report

CP in Motion

Child's name

This report was prepared on the basis of a gait analysis that was carried out as part of the research project: "Individual multidisciplinary intervention". The report is prepared in English, but also includes a short summary in Danish containing the most important findings.

The report describes:

- Body functions and structure impairments that are believed to affect walking.
- The elements of the child's gait (Features), where the movement pattern differs from the movement pattern in children without disabilities. The elements are documented as deviations in the course of the curve over movements of the pelvis, hip, knee and ankle.
- Additional information used in the interpretation of the survey results (e.g. physical examination, functional test and questionnaires).

The report

The report contains the following sections:

- Relevant history and other measures
- Child's height, weight, walking speed, stride cadence, font length (Orientation)
- Gait index describing movements' deviation from gait in children without disabilities (GDI, GPS and GVS).
- Description of the quality of the study (Quality)
- Comparison of study findings and supplementary information, as well as interpretation of impairments affecting time (Evidence and interpretation)
- Graphs of the child's movements during the course of marking survey findings (Gait Data and Consistency plots)
- Description of survey findings (Description)
- The physical examination and information on walking speed, stride length etc. (Physical examination and temporal-spatial parameters).
- Results of functional tests and questionnaires (Other measures)

The report will be conferred interdisciplinary as part of a research project. Thereafter, on the basis of the examination findings, an overall description of relevant contributions that health personnel are recommended to consider, will be prepared.

Physiotherapist Helle Mätzke Rasmussen

Gait Analysis Laboratory – The Othorpedic Research Unit, University of Southern Denmark & Odense University Hospital

Kontact: xxx@xxx.xx / +45 xx xx xx xx

Projekt ID	xxxx
Dato	xx-xx-xxxx

Gait analysis report

CP in Motion

Patient details

**Name**	xxx	**Age**	x	**Date**	xx-xx-xxxx
**Diagnose**	Unilateral CP, right side	**GMFCS**	II	**FMS**	5 – 5 – 5

Findings

**The most likely impairments affecting the walking patter are:**		
**Impairment**	**Evidence**	**Effect on walking**
- Reduced ROM in ext. in right knee	Clear	Major
- Reduced strength around right ankle	Clear	Major
- Reduced ROM in dorsal flexion on right	Probable	Moderate
- Leg length discrepancy	Possible	Minor
**Comments**<br>- All gait features have been attributed to the impairments listed above.<br><br>Video analysis with orthotics<br>- In the video where the child walks with drop foot brace / orthotics, gait is more secure and faster. Furthermore the child walks with heel strike on both feet.		

Notes on reading this report

The rest of this report outlines the evidence in support of the findings listed above. The Orientation and Quality sections should be read first as they may contain information that is pertinent to how the data is interpreted. For each of the impairments identified there is a box in the Evidence section that lists gait features and elements of the clinical exam and other data that have been linked to that specific impairment. The final section outlines the symbols used to mark up specific features on the gait graphs. The report should be read alongside a version of the data on which the features have been marked.

The report is based on the first of two clinical gait analysis, that have been performed as part of a research project, that is conducted at the Gait Analysis Laboratory by physiotherapist Helle Mätzke Rasmussen.

History

**Relevant history**	The child is followed in the Cerebral Palsy Follow-up program at XXX and by PT XXX<br>The child walks with a drop-foot orthotic, that he/she is pleased with.<br>The child has a lumbar scoliosis.<br>The child's mother tells that the child's goal is to learn to ride a 2-wheel bicycle.
**Other measures**	1-min walk test: 80 meter<br>GMFM-66 Score: 80 point 75,4-84,6 (95% CI)<br>PODCI Global functioning Scale: 73 (1-100)<br>PEDI: Functional skills: 56 (0-59), Caregiver assistance: 32 (0-35)<br>PedsQL: 44 – 70 (0-100)<br>Fatique / Speech and communication 44<br>Movement and balance: 70<br>MPOC-20: 3,3 – 4,6 (0-7)

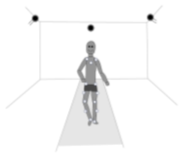

Projekt ID	xxxx
Dato	xx-xx-xxxx

Orientation

Height	1,30 m	Speed	90 %	Step length (L)	85 %
Weight	26 Kg	Cadence	93 %	Step length (R)	91 %
BMI	15	Spatial gait parameters and expressed as % of those for children with no neuromuscular pathology (not normalised to leg length).			
**Gait Indices**					
GDI Left and right	70 76	GDI Overall	73	GVS scores	Left Right / Overall
GPS Left and right	11,4 9,9	GPS Overall	11,4	Pelvic Ant tilt	4,0
Motion Analysis Profile				Hip flex	11,9 8,9
				Knee flex	16,7 19,4
				Ankle flex	8,7 6,3
				Pelvic obl up	7,6
				Hip add	8,4 7,6
				Pelvic int rot	5,3
				Hip int rot	18,6 13,0
				Foot prog	9,2 6,5

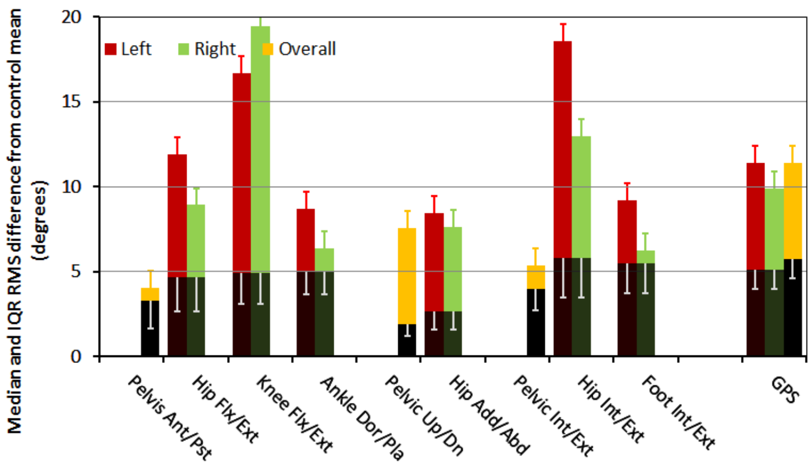

Comments on video

- The child stands with weight bearing primarily on her left leg and with the right leg in slight flexion. Leg length discrepancy is visual on the frontal video in stance, in both knee and pelvic. Visually it seems more pronounced than the measured 1.5 cm.
- The child walks a bit unsecure, which is seen in the end of the frontal video, where she/he changes direction and loses balance. The child walks with the leg in adduction and often crosses the midline. The child holds his/her right arm in a position with light flexion in the elbow. When the child walks with shoes and orthotics, gait is more secure and faster. Furthermore the child walks with heel strike.

Quality

Is the data likely to be representative of the subject's usual walking pattern?

- The child's mother confirms that the walking pattern is representative of the normal walking pattern

Are there any concerns regarding consistency of traces?

- No, but there is some inconsistency in the transverse plane.

Is there any evidence of measurement artefact or inconsistency in the data?

- No

Evidence and interpretation

**Impairment:** Reduced ext. ROM in right knee	Evidence: Clear	Effect on walking: Major
**Impairment:** Leg length discrepancy	Evidence: Possible	Effect on walking: Minor

Features:

- **h** Abnormal slope in knee extension right
- **a** Too much anterior pelvic tilt
- **d** Too little hip extension (right)
- **j** Too little right dorsiflexion in midstance
- **e** Too much right hip flexion in swing
- **f** Too much right hip ext. rotation in swing
- **l** Increased int. foot progression
- **b** Decreased pelvic obliquity (right down)
- **c** Increased right pelvic ext. rotation

**Supplementary data:**	Left	Right
Knee extension	0°	-8°
Quadriceps lag (difference)	0°	0°
Knee ext. strength	5	4+
Leg length	63.5	62.0

Comments:

Too much anterior pelvic tilt (a), too little hip ext. in stance (d) and too little right dorsiflexion in stance (d) might be a compensatory effect of the lack of knee extension on right (h).

To much right hip flexion (e) and ext. rotation in swing (f) might be to clear left foot, in compensation for the flexion in the right leg (h, d, i) in stance and leg length discrepancy. To keep the foot aligned the foot is internal rotated (l). Furthermore the decreased pelvic obliquity (b), and increased right pelvic ext. rotation (c) might compensation for the difference.

**Impairment:** Reduced strength around right ankle	Evidence: Clear	Effect on walking: Major
**Impairment:** Reduced range of movement right	Evidence: Probable	Effect on walking: Moderate

Features:

- **k** Too little dorsiflexion in swing and ini. contact
- **i** Too much left knee flexion in early stance
- **j** Too little right dorsiflexion in midstance
- **c** Increased pelvic rotation
- **g** Too much knee flexion in late swing and initial contact.

**Supplementary data:**	Left	Right
Plantar flexor spasticity	15°	5°
Dorsiflexion (knee 90°)	15°	8°
Dorsiflexion (knee 0°)	20°	10°
Dorsi flexor strength	5	4
Plantar flexor strength	5	4

Comments:

Too little dorsiflexion in swing (k) is seen due to reduced strength in dorsiflexion, which causes the increased knee flexion in late swing (i). The decrease in dorsiflexion in midstance (j) might also be due to decreased range of movement in dorsiflexion (8° / 10°).

Increased pelvic rotation (c) and increased knee flexion in early stance (g) might also be a compensatory effect for decreased push-off in plantar flexion (plantar flexor strength).

Projekt ID	xxxx
Dato	xx-xx-xxxx

Gait data

Pelvic Tilt

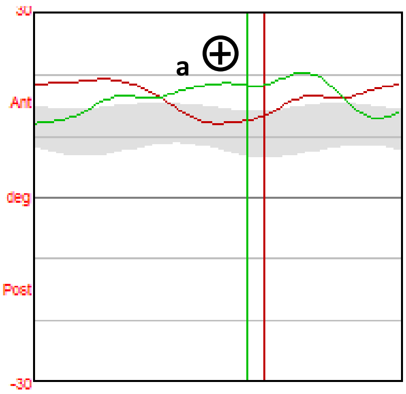

Pelvic Obliquity

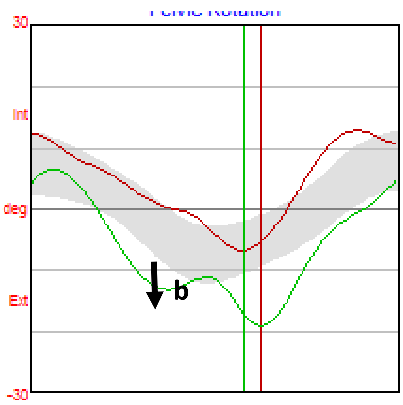

Pelvic Rotation

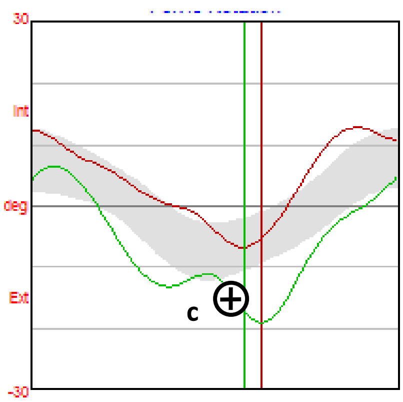

Hip Flexion

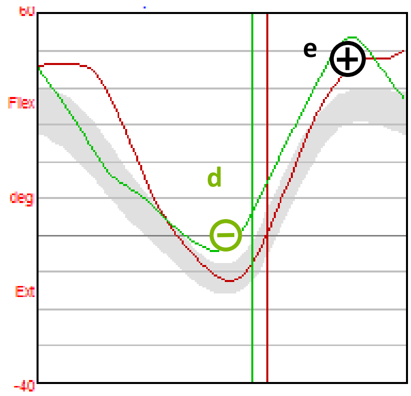

Hip Adduction

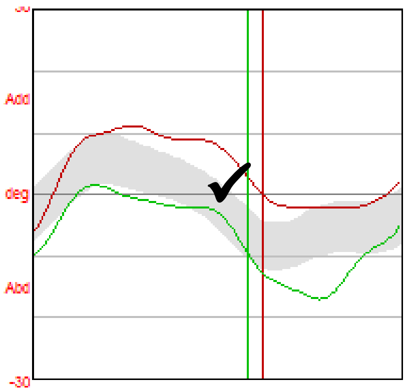

Hip rotation

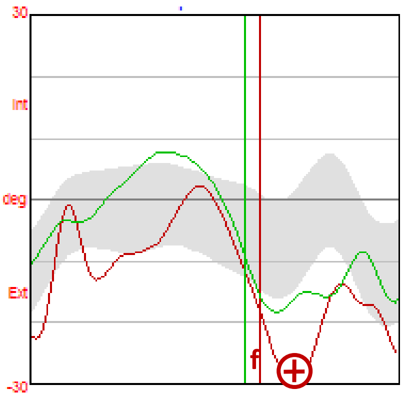

Knee Flexion

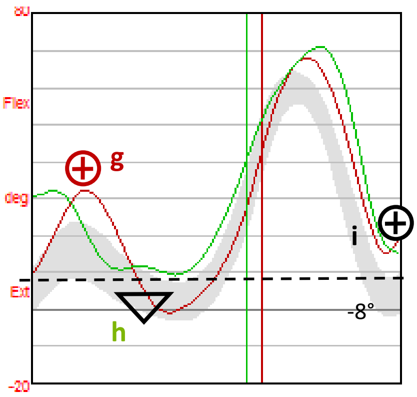

Dorsiflexion

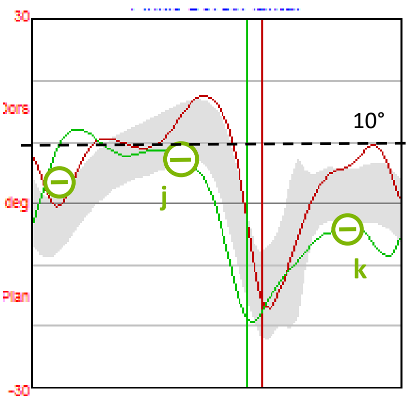

Foot Progression

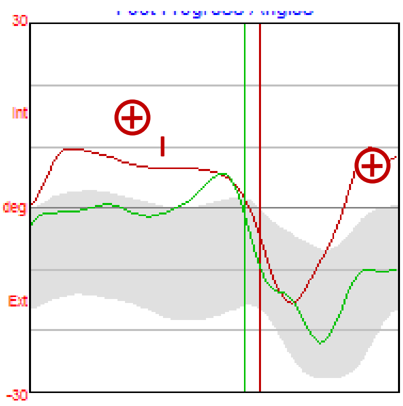

Projekt ID	xxxx
Dato	xx-xx-xxxx

Consistency plots

Pelvic Tilt

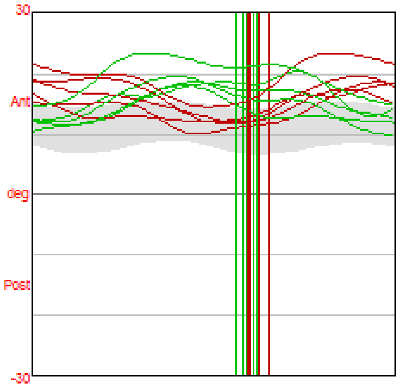

Pelvic Obliquity

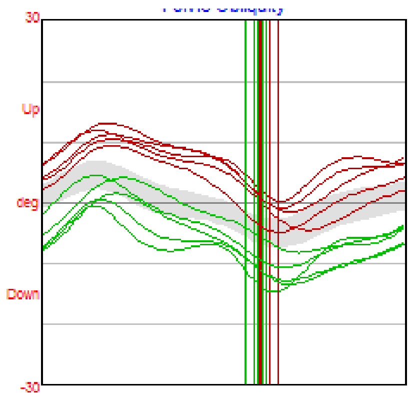

Pelvic Rotation

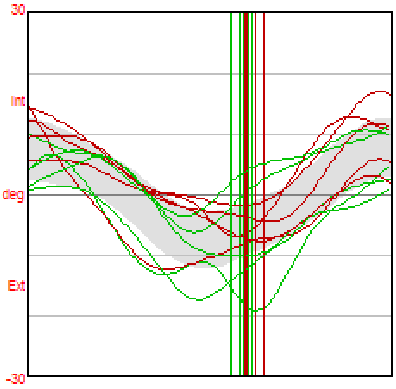

Hip Flexion

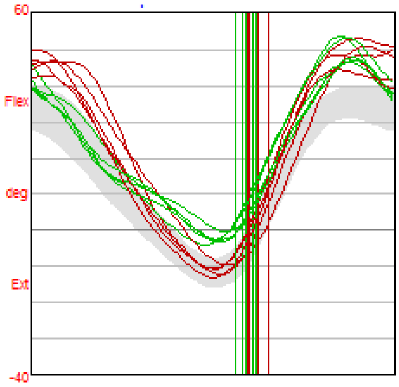

Hip Adduction

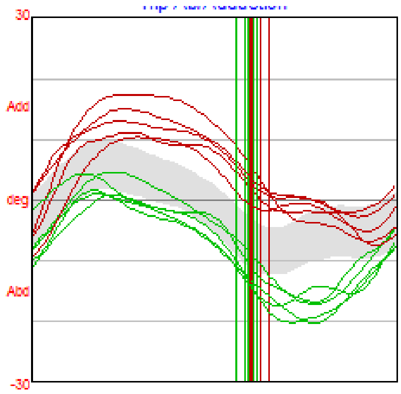

Hip rotation

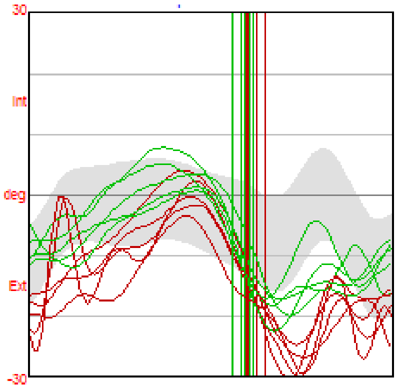

Knee Flexion

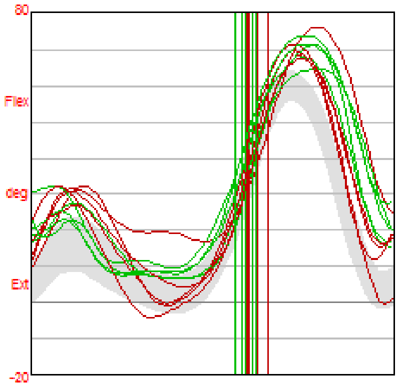

Dorsiflexion

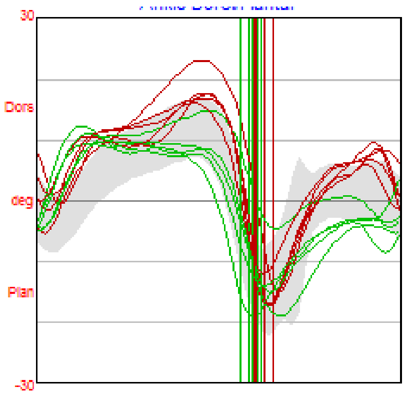

Foot Progression

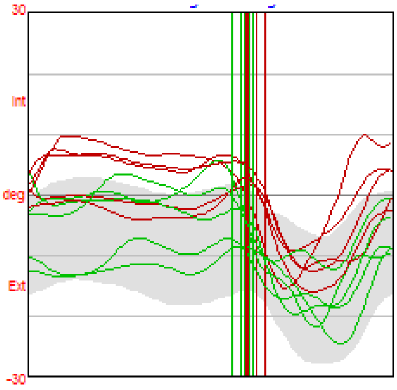

Description

Letter	Type	Side	Variable	Timing
a	Too much	Right	Pelvic ant tilt	Late stance and initial swing
b	Decreased	Right	Pelvic up obliquity	All of cycle (Right: down, left up)
c	Increased	Right	Pelvic ext. rotation	Late stance and swing (inconsistent)
d	Too little	Left	Hip extension	Initial stance and late swing
e	Too much	Both	Hip flexion	Late swing and initial stance (left)
f	Too much	Right	Hip ext. rotation	Swing
g	Too much	Left	Knee flexion	Early stance
h	Abnormal slope	Right	Knee flexion	Mid-stance
i	Too much	Both	Knee Flexion	Late swing and initial contact
j	Too little	Right	Dorsiflexion	Midstance
k	Too little	Right	Dorsiflexion	Swing and initial contact
l	Too much	Right	Foot progression	Stance and late swing (inconsistent)

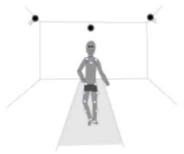

Projekt ID	xxxx
Dato	xx-xx-xxxx

Physical examination and temporal-spatial parameters

Hip	Left	Right
Hip ext. range (Thomas test)	12°	15°
Hip flexor strength	5	4+
Hip ext. strength	5	4+
Hip abd. range (hip 0° knee 0°)	45°	45°
Hip abd. range (hip 0° knee 90°)	45°	45°
Hip abd. strength	5	4+
Hip add. tone (MAS)	-	-
Hip int. rotation range	40°	50°
Hip ext. rotation range	40°	35°

Knee	Left	Right
Mod. Popliteal angle (m. flex hip)	150°	155°
True popliteal angle	150°	140°
Hamstring shift	0°	15°
Hamstring spasticity (Tardieu)	-	-
Hamstring spasticity (MAS)	-	-
Knee extension	0°	-8°
Quadriceps lag (active)	0°	-5°
Quadriceps lag (passive)	0°	-5°
Knee extensor strength (90-20° flex)	5	4+
Rectus length (Ely)	140°	140°
Rectus spasticity (Fast Ely – Tardieu)	-	-
Rectus spasticity (Fast Ely – MAS)	-	-
Knee flex. strength	5	4+

Ankle	Left	Right
Dorsiflexion (knee 90°) Soleus	15°	8°
Dorsiflexion (knee 0°) Gastroc.	20°	10°
Plantar flexor tone (MAS)	-	-
Plantar flexor spasticity (Tardieu)	15°	5°

	Left	Right
Plantar flexor strength	5	4
Dorsi flexor strength (knee 90°)	5	4
Inventor strength	4	3
Evertor strength	4	3
Confusion test	-	+

Other measure	Left	Right
Tibial torsion	6°	20°
Posture of feet	-	-

	Left	Right
Leg length (cm)	63,5	62,0
Knee valgus / varus *(if relevant)*	-	-

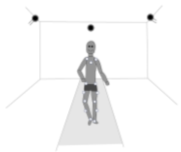

Projekt ID	xxxx
Dato	xx-xx-xxxx

Tibial torsion and foot alignment

Left

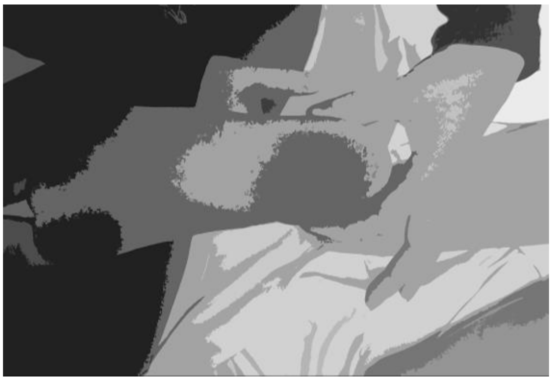

Tibial torsion	6°
Thigh-hindfoot angle	2°

Right

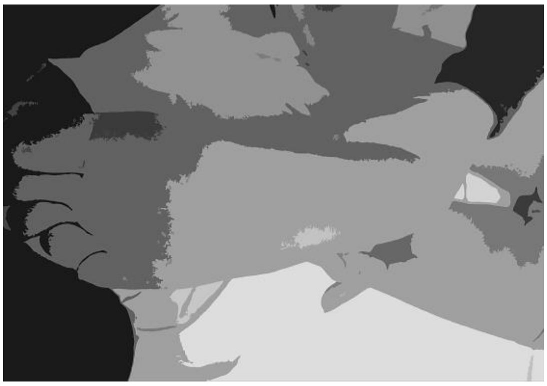

Tibial torsion	20°
Thigh-hindfoot angle	22°

Spatial gait parameters

Walking speed	1.22 m/s
Cadance	129.5 Step/min
Stride length	1.05 m
Left step length	0.45 m
Right step length	0.48 m
Left step with	0.06 m
Right step with	0.05 m

Walking speed	93 % normal
Cadance	91 % normal
Stride length	97 % normal
Left step length	85 % normal
Right step length	91 % normal
Left step with	62 % normal
Right step with	91 % normal

Projekt ID	xxxx
Dato	xx-xx-xxxx

Abbreviation

Na	Not available
Ia	Nothing abnormal
GMFM-66	Gross Motor Function Measure, 66 item version
PODCI	Pediatric Outcomes Data Collection Instrument
PEDI	Pediatric Evaluation Disability Inventory
MPOC-20	Measure of Processes of Care, 20 item version

Feature symbols

↑	Increased (all of cycle)	↓	Decreased (all of cycle)	Arrow ending on trace
⊕	Too much (part of cycle)	⊖	Not enough (part of cycle)	On trace (or just above or below feature)
←	Too early	→	Too late	Arrow ending on feature
↔	Too long	→←	Too short	Arrow spanning feature
⌶	Increased range	⫫	Decreased range	Adjust height and width as appropriate
◿	or	◺	Abnormal slope	Sloping side along feature
✔	Within normal limits			Only use if particular reason for doing so
?	Possible artefact			On feature
⬭	Other feature			Encircling feature

Other measures

**GMFM-66**	**GMFM Score estimated with Gross Motor Ability Estimator 2** (0-100)<br><br>19 item tested - GMFM-66: 80 point<br>95% Confidence interval: 75,4 – 84,6
**PODCI**	**PODCI standardized mean subscales** (0-100)<br><br>Global functioning Scale: 73<br>Upper Extremity Scale: 75<br>Transfer & Basic Mobility Scale: 95<br>Sports and Physical Functioning Scale: 72<br>Pain/Comfort: 49<br>Happiness: 70
**PEDI**	**PEDI Subscale: Functional mobility** (0-59 / 0-35)<br><br>Functional skills: 56<br>Caregiver assistance: 32
**PedsQL**	**PedsQL Subscales** (0-100)<br><br>Daily Activities: 67<br>School Activities: 56<br>Movement and Balance: 70<br>Pain and Hurt: 56<br>Fatigue: 44<br>Eating Activities: 65<br>Speech and Communication: 44
**MPOC-20**	**MPOC-20 Categories** (0-7)<br><br>Enabling and Partnership: 3,3<br>Providing General Information: 4,2<br>Coordinated and Comprehensive Care for the Child and Family: 3,7<br>Providing Specific Information about the Child: 3,8<br>Respectful and Supportive Care: 4,6

Interdisciplinary interventions

CP in Motion

'Child's name'

This report was prepared on the basis of a gait analysis that was carried out as part of the research project: "Individual multidisciplinary intervention". The report is prepared in English, but also includes a short summary in Danish containing the most important findings (*this section has been removed*).

The report describes:

- Body functions and structure impairments that are believed to affect walking.
- The elements of the child's gait (Features), where the movement pattern differs from the movement pattern in children without disabilities. The elements are documented as deviations in the course of the curve over movements of the pelvis, hip, knee and ankle.
- Additional information used in the interpretation of the survey results (e.g. physical examination, functional test and questionnaires).

Multidisciplinary recommendations

The recommendations have been made based on a review of the report on xx-xx-xxxx by an interdisciplinary team:

The multidisciplinary team consisted of:

- Chief Physician, Clinical Associate Professor XXXXXXXXX, Orthopedic Surgery Department, Odense University Hospital
- Chief Physician, XXXXXXXXX, H.C. Andersen Children's Hospital
- Associate Professor, XXXXXXXXX, Orthopedic Surgery Research Unit, Odense University Hospital and University of Southern Denmark
- Physiotherapist, Ph.D. student XXXXXXXXX, Orthopedic Surgery Research Unit, Odense University Hospital and University of Southern Denmark

The recommendations describe which functional impairments affect the child's gait pattern and what interventions that the child, parents and health professionals should consider.

Projekt ID	xxx
Dato	xx-xx-xxxx

Patient

**Name**	xxx	**Age**	x	**Date**	xx-xx-xxxx
**Diagnose**	Unilateral CP, right side	**GMFCS**	II	**FMS**	5 – 5 – 5

Findings

Detailed description

The study on xx-xx-xxxx in the Gait Analysis Laboratory shows that the gait pattern, i.e. the way the child moves his/her joints in the legs during walking is affected by the following:

- Reduced joint mobility in the right knee, which is not fully extended.
- Reduced muscle strength around the right ankle
- Reduced joint movement in right foot lift (dorsal flexion)
- Leg length difference (measured at x cm for the study)

Furthermore, the examination shows that when the child walks with drop foot brace / orthotics, gait is more secure and faster. The child walks with heel strike on both feet.

The child attends CPOP controls at the XXX Hospital.
A right convex scoliosis has been observed.

Intervention recommendations

Interventions based on the observed functional impairments

Based on the findings of the gait study, the following actions are recommended:

Reduced joint mobility in right knee and leg length difference

- Orthopedic surgical assessment of efforts to improve joint mobility of the right knee joint, and assessment of the leg length difference.

Reduced strength around the right ankle joint

- Exercises to improve muscle function in lifting the forefoot (dorsal flexion) and heel lift (plantar flexion) on the right leg so as to improve lifting of the forefoot in the swing phase and at foot contact during walking.
- Continuous use of the drop foot brace/orthotics.

Reduced joint movement in the right foot lift

- Exercise efforts to maintain muscle length around the right ankle, so as to maintain current joint mobility

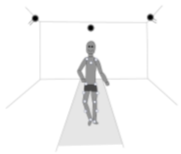

Projekt ID	xxx
Dato	xx-xx-xxxx

Detailed description for health professionals

Interventions based on the observed functional impairments

Based on the findings of the gait study, the following actions are recommended:

Reduced joint mobility in right knee and leg length difference

- Orthopedic surgical assessment of efforts to maintain and improve joint mobility of the right knee joint, as well as assessment of leg length discrepancy (note, the child has a right convex lumbar scoliosis).
- At the interdisciplinary conference various operations were discussed, but it is agreed that a precise recommendation must depend on the clinical examination and possibly supplementary examinations. The orthopedic surgeon is asked to make a decision on the further plan, as well as inform the family of the need for further examinations and treatment options.

Reduced strength around the right ankle joint

- Exercises to improve muscle function in lifting the forefoot (dorsal flexion), with flexed hip and knee (as in the middle of the swing phase) as well as with a stretched knee (as at the end of the swing phase), where the right foot is held at an approx. 5-10 degree plantar flexion. The action should be primarily focused on concentric and isometric muscle strength in the tibialis anterior with the flexed and extended knee.
- Exercise efforts to improve muscle function in heel lift (plantar flexion) on the right leg to improve push-off. The training should have a special focus on plantar flexion with extended hip and knee, as at the end of the standing phase.

Reduced joint movement in the right foot lift

- Exercise efforts to maintain muscle length around the right ankle, so as to maintain current joint mobility
